# Removal of Wall-Adherent Inferior Vena Cava Thrombus with a Combined Approach Using Vacuum-Assisted Thrombectomy and a Rotational Thrombectomy Device

**DOI:** 10.1155/2023/5178998

**Published:** 2023-08-18

**Authors:** Julius Kaemmel, Roland Heck, Pia Lanmüller, Volkmar Falk, Christoph Starck

**Affiliations:** ^1^Department of Cardiothoracic and Vascular Surgery, Deutsches Herzzentrum der Charité (DHZC), Augustenburger Platz 1, 13353 Berlin, Germany; ^2^Charité-Universitätsmedizin Berlin, Freie Universität Berlin, Humboldt-Universität zu Berlin, and Berlin Institute of Health, Augustenburger Platz 1, 13353 Berlin, Germany; ^3^DZHK (German Centre for Cardiovascular Research), Berlin, Germany; ^4^Berlin Institute of Health at Charité-Universitätsmedizin Berlin, Berlin, Germany; ^5^Translational Cardiovascular Technologies, Institute of Translational Medicine, Department of Health Sciences and Technology, Swiss Federal Institute of Technology (ETH) Zurich, Zurich, Switzerland

## Abstract

*Introduction*. Large thrombi in the inferior vena cava pose a high risk for a pulmonary embolism. Percutaneous extracorporeal circulation-based vacuum-assisted thrombus aspiration is a viable option for removal. Wall adherence of thrombotic material can compromise procedural success. *Case Report*. A 46-year-old female presented with a subtotal thrombotic occlusion of the inferior vena cava and the proximal right common iliac vein after weaning from extracorporeal life support. Due to severe wall adherence of the thrombotic material, the patient was treated with the combination of percutaneous extracorporeal circulation-based thrombus aspiration using the AngioVac system and a rotational thrombectomy device.

## 1. Introduction

Extracorporeal life support-associated thrombosis in the deep venous system is a known complication [1]. Large thrombi in the inferior vena cava pose a high risk for a pulmonary embolism and are an indication of thrombectomy or lysis [2, 3]. Percutaneous extracorporeal circulation-based thrombus aspiration with the AngioVac system has proved to be a feasible alternative to surgical removal or medical thrombolysis [4]. However, wall-adherent thrombi can compromise the procedural success of vacuum-assisted thrombectomy. This case report describes a strategy for the removal of a large thrombus extending from the proximal right common iliac vein through the entire inferior vena cava (IVC) through a combination of extracorporeal circulation-based percutaneous vacuum-assisted thrombectomy and rotational thrombectomy to increase the procedural success rate in wall-adherent central deep vein thrombosis.

## 2. Case Report

**Figure 1:**
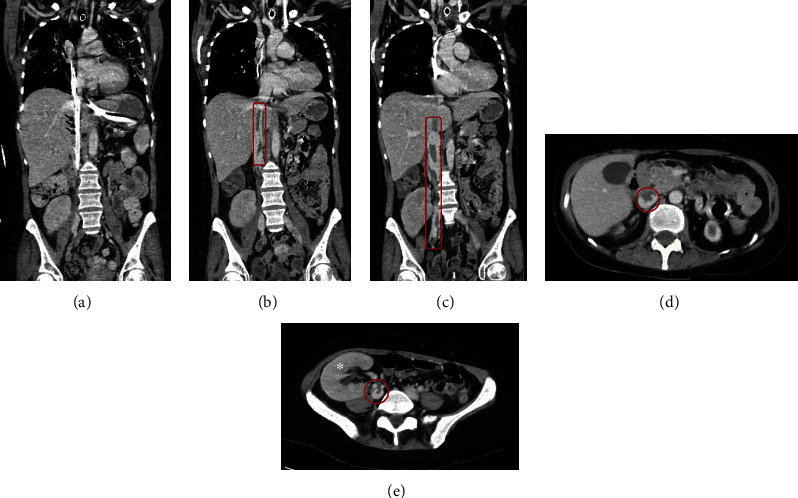
Preoperative computed tomography angiography (CTA) confirming the diagnosis of inferior vena cava and right proximal iliac vein thrombosis. (a) The black arrows mark the ECMO cannula prior to decannulation localized in the inferior vena cava. (b, c) After removal of the venous ECMO cannula, CTA shows extensive thrombosis of the inferior vena cava and the proximal right iliac vein as highlighted by the red box. (d) Subtotal occlusion of the inferior vena cava at the level of the liver is highlighted by the red circle. (e) Subtotal occlusion of the vessel lumen next to the heterotopic kidney transplant (^∗^ marking the kidney transplant).

A 46-year-old female was admitted due to a subtotal thrombotic occlusion of the entire IVC and the proximal right common iliac vein after decannulation from extracorporeal life support which was confirmed by computed tomography angiography (CTA) (Figure 1). Prior to admission, the patient had been treated in a medical intensive care unit for severe COVID-19 pneumonia and required veno-venous extracorporeal membrane oxygenation (vv-ECMO) for 36 days. The patient was status post kidney transplant under immunosuppressive therapy. Prior to vv-ECMO initiation, the patient was on prophylactic anticoagulation with heparin. Once ECMO support was established, anticoagulation was intensified with unfractionated heparin with a therapeutic partial thromboplastin time (PTT) target. Due to the extensive thrombosis with subtotal occlusion of the inferior cava and right iliac vein and the risk for pulmonary embolization, the patient was allocated to mechanical thrombectomy after a multidisciplinary decision-making process.

On presentation, the patient was hemodynamically stable without the need for catecholamines and required only low-flow oxygen supplementation via an existing tracheostomy. The patient was transferred directly to the hybrid operating room.

After induction of general anesthesia, a 6 French (F) sheath was placed in the right internal jugular vein. Preoperative transesophageal echo (TEE) showed good biventricular function and only mild tricuspid regurgitation.

**Figure 2:**
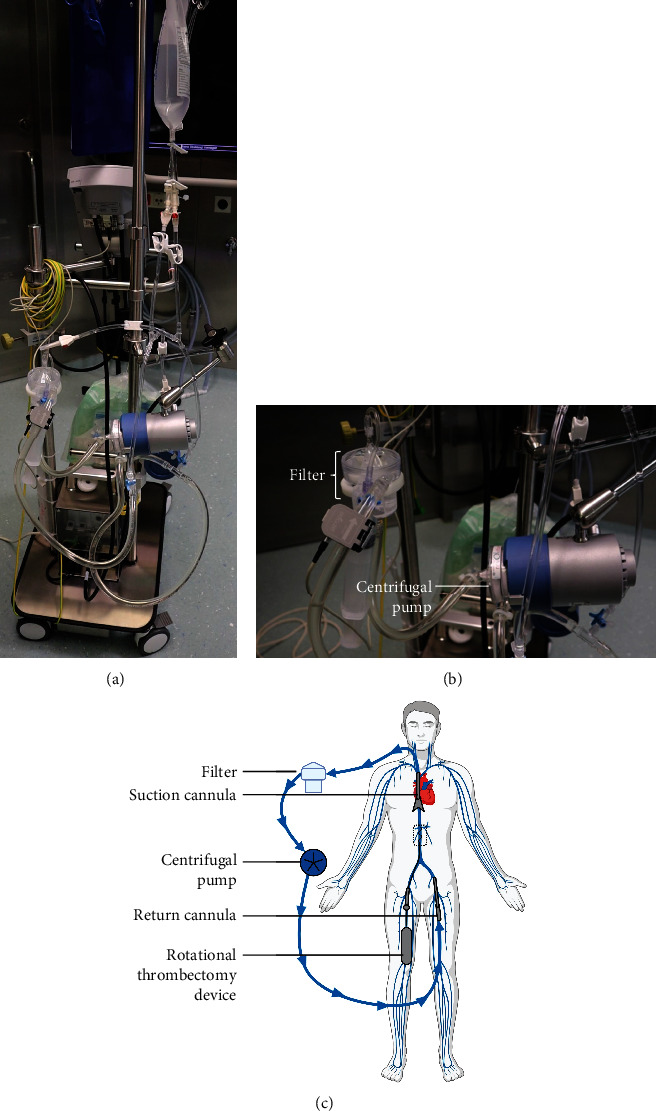
Setup of the AngioVac system. (a) Depicted is the primed extracorporeal circuit prior to connection to the patient. (b) Focused view of the filter and the centrifugal pump. (c) Schematic illustration of the application of the AngioVac system in combination with the Cleaner 15™ rotational thrombectomy device. In this case, due to thrombus formation in the inferior vena cava and the right iliac vein, the suction cannula was introduced into the right jugular vein, and venous return was administered via the left femoral vein. The rotational thrombectomy device was introduced via the right femoral vein. The dotted area shows the released sinusoidal wire of the thrombectomy device just distally of the AngioVac suction cannula.

As described in detail by Haupt et al. [5], the veno-venous extracorporeal circuit used at our institution for percutaneous aspiration procedures consists of the 22 F AngioVac (Gen 3) 20° suction cannula (AngioDynamics, Latham, NY, USA) for venous drainage, a CAPIOX® BT15 bubble trap (Terumo Cardiovascular, Ann Arbor, MI, USA) as a filter for aspirated thrombotic material, a centrifugal pump (Revolution 5, LivaNova, Mirandola, IT), and a 16 F Edwards Fem-Flex II cannula (Edwards Lifesciences, Irvine, CA, USA) for venous return. During the procedure, heparin is used for anticoagulation with a target-activated clotting time of 250 to 300 seconds. During these procedures, the negative pressure created at the tip of the aspiration cannula ranges between -2 mmHg in phases of recirculation and above -300 mmHg in the moment of aspiration of thrombotic material [5]. The setup used in this case is shown in Figure 2.

**Figure 3:**
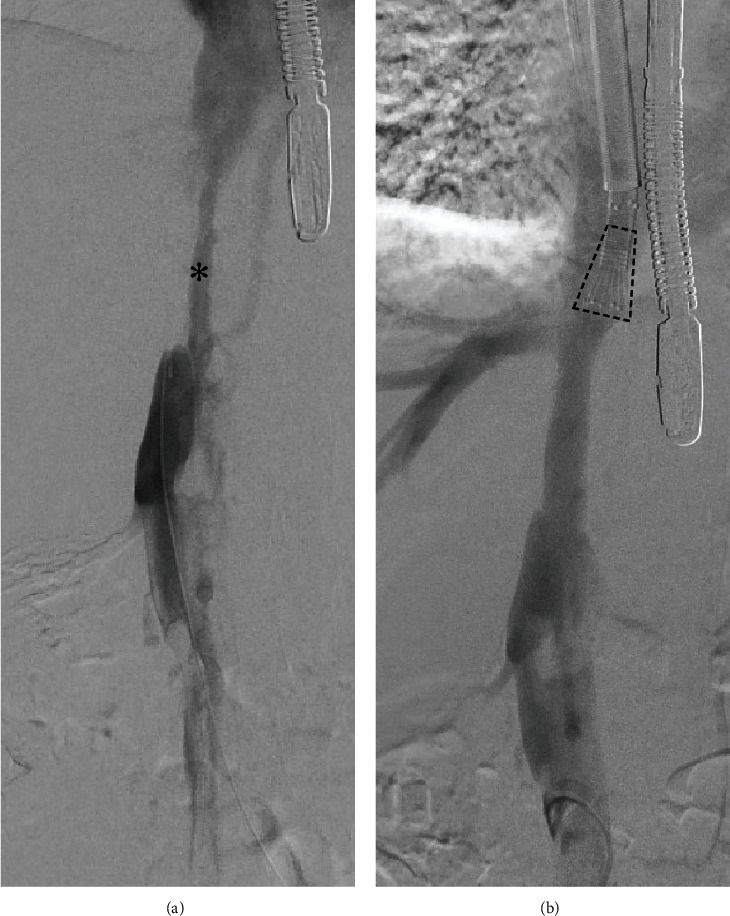
Pre- and postinterventional venogram of the inferior vena cava. (a) Preoperative venogram shows a subtotal occlusion of the inferior vena cava. The asterisk marks the remaining lumen of the IVC. (b) After the procedure, the lumen of the inferior vena cava is restored. The dotted cone shows the tip of the suction cannula.

As a first step, the right femoral vein was cannulated percutaneously with an 8 F sheath, followed by venography of the IVC via the same access. In line with preoperative diagnostic studies, the venogram revealed a subtotal thrombotic occlusion of the IVC and the proximal right common iliac vein (Figure 3(a)).

After systemic heparin administration, a 16 F Edwards Fem-Flex II cannula (Edwards Lifesciences, Irvine, CA, USA) was introduced into the left femoral vein for venous return using Seldinger's technique. The 6 F sheath in the right jugular vein was exchanged via an Amplatz Ultra Stiff guidewire (Cook Medical, Bloomington, IN, USA) for a 26 F Gore Dry Seal sheath (W.L. Gore & Associates, Inc., Flagstaff, AZ, USA). As a last step prior to initiation of the extracorporeal circuit, the 22 F AngioVac (Gen 3) 20° angled tip cannula (AngioDynamics, Latham, NY, USA) was introduced via the 26 F sheath, and both the return cannula and the AngioVac suction cannula were connected to the extracorporeal circuit.

**Figure 4:**
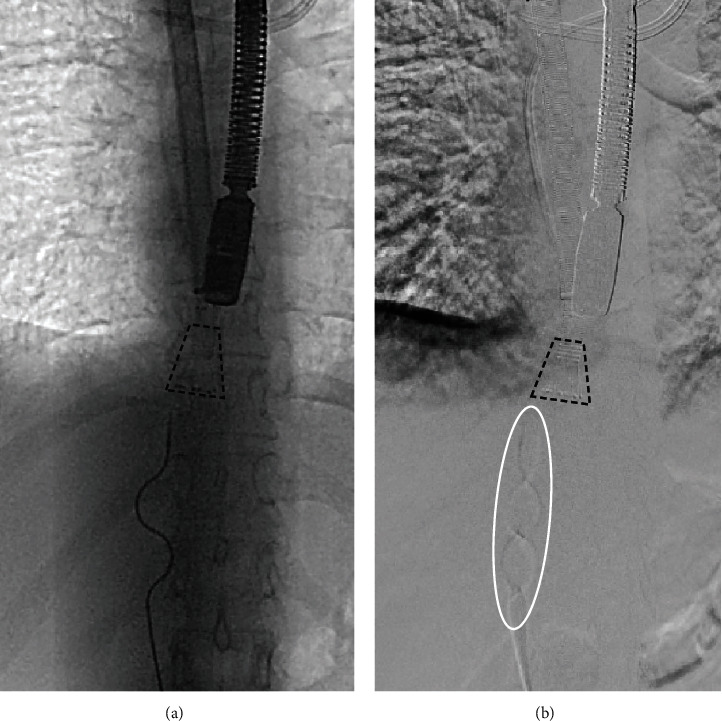
Mobilization of wall-adherent thrombus with the rotational thrombectomy device. (a) The funnel at the tip (dotted cone) of the AngioVac cannula is positioned just proximal to the tip of the Cleaner 15™ thrombectomy device. Note that the sinusoidal wire has been released from the catheter of the rotational thrombectomy device. (b) Under continuous aspiration of the AngioVac system, the rotational thrombectomy device is engaged, and the sinusoidal wire rotates within the vessel. The white ellipsoid shows the rotating action of the sinusoidal wire.

**Figure 5:**
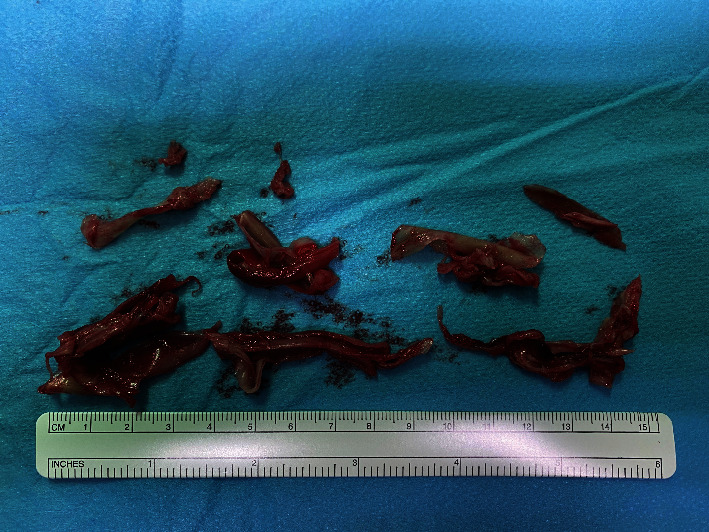
Thrombotic material found in the filter of the extracorporeal circuit after the procedure. Pathologic examination showed that the aspirated thrombus material consisted of fibrin with a moderate amount of erythrocytes. No bacterial growth was detected during microbiological testing.

The extracorporeal circulation was started, and thrombotic material in the IVC and the proximal right common iliac vein was aspirated under continuous monitoring with TEE and fluoroscopy. Due to severe wall adherence of large portions of the thrombotic material, a rotational thrombectomy system Cleaner 15™ (Argon Medical Devices, Plano, TX, USA) was introduced via the 8 F sheath in the right femoral vein. The tip of the AngioVac cannula was placed just cranially to the tip of the released sinusoidal wire of the rotational thrombectomy device (Figure 4(a)). The thrombectomy system was engaged under continuous aspiration, and the wall-adherent thrombus in the IVC and the right iliac vein were mobilized (Figure 4(b)) and simultaneously evacuated. After the thrombotic material was safely removed (Figure 5), the extracorporeal circulation was discontinued.

In order to evaluate the procedural success, another venography was performed which showed almost complete removal of the thrombotic material (Figure 3(b)). The last steps comprised the removal of the cannulas and the sheaths, followed by the closure of the skin incisions with sutures.

Postprocedural TEE showed no change in ventricular contractility, no new valvulopathy, and no signs of pulmonary embolism.

Completion of the procedure was followed by weaning from anesthesia. Once the patient resumed spontaneous breathing under low-flow oxygen supplementation, she was transferred back to the medical ICU with stable hemodynamics. The total duration of the surgical procedure was 68 min, and the patient was mechanically ventilated for 150 min. After the procedure, the patient received therapeutic anticoagulation with low-molecular-weight heparin. During the further postoperative course, the patient was switched to therapeutic anticoagulation with a direct oral anticoagulant (DOAC). By her discharge on the 19th postoperative day, the patient had recovered well and was transferred to a rehabilitation facility in hemodynamic and respiratory stability. Therapeutic anticoagulation was indicated for at least 3 to 6 months after discharge.

## 3. Discussion

Vacuum-assisted thrombectomy using the AngioVac device is a well-established technique for debulking large cardiac implantable electronic lead vegetations and tricuspid vegetations in endocarditis [6–8]. Furthermore, it presents a therapeutic option for the treatment of venous thrombosis of central or iliac veins and even pulmonary embolisms [9]. A well-known cause of procedural failure of mechanical and vacuum-assisted thrombectomy is the adherence of chronic thrombus to the vessel wall [4, 10]. This issue can be overcome by using rotational thrombectomy devices such as the Cleaner 15™ since its adjunctive use increases the procedural success in pharmacomechanical thrombectomy. In the case of the Cleaner 15™ rotational thrombectomy device, a sinusoidal wire rotating at 4,000 rpm macerates the thrombus while simultaneously detaching it from the vessel wall [11]. Due to the potential danger of dislodgement of thrombotic material and pulmonary embolisms, these procedures are often performed with a vena cava filter in place [11, 12]. Despite being a known technique for thrombectomy in smaller veins such as the femoral veins or AV-fistulas, rotational thrombectomy is not yet established during the use of vacuum-assisted thrombectomy of wall-adherent thrombi in the IVC [13–15].

In the presented case, due to severe wall adherence of the thrombus, vacuum-assisted thrombectomy with the AngioVac device alone did not offer sufficient procedural success. However, the use of the AngioVac allowed for the adjunctive use of the Cleaner 15™ rotational thrombectomy system, since mobilized debris could be aspirated and did not pose a risk of pulmonary embolism in this setting. The combined approach allowed for complete recanalization of the inferior vena cava and the right iliac vein.

The use of a rotational thrombectomy device during percutaneous vacuum-assisted thrombectomy may be a useful addition for the removal of large wall-adherent thrombi in the inferior vena cava and the proximal iliac veins. This technique potentially increases the procedural success rate in patients presenting with chronic wall-adherent thrombi and might be useful in preventing further escalation of surgical therapy.

## Data Availability

The data used to support the findings of this study are included within the article.

## Supplementary Materials

Supplementary Materials Supplementary material file with a description of key technical aspects for performing the described procedure.
