# Supplementary material for: Removal of Wall-Adherent Inferior Vena Cava Thrombus with a Combined Approach Using Vacuum-Assisted Thrombectomy and a Rotational Thrombectomy Device
Source: Case Rep Vasc Med. 2023 Aug 18;2023:5178998. doi: 10.1155/2023/5178998 (PMC10457166; doi:10.1155/2023/5178998)
Supplement: Supplementary Materials — Supplementary material file with a description of key technical aspects for performing the described procedure. [file 5178998.f1.docx]

Supplementary material:

**Key technical aspects:**

- In case of inferior vena cava thrombosis, use the AngioVac circuit in a right internal jugular to femoral vein configuration.
- In case of iliac vein involvement introduce the rotational thrombectomy device via the femoral vein on the side of the thrombosis.
- Position the AngioVac drainage cannula distally from the right atrium in order to prevent pulmonary embolization of thrombotic material during rotational thrombectomy in the distal inferior vena cava.
- The aspiration cannula should only be positioned under echocardiographic and fluoroscopic control in order to avoid injury to the heart and the vessel walls.
- Once the suction is initiated, only micromovements should be applied on the drainage cannula.
- As a safety measure in hemodynamic unstable patients consider arterial access in addition to the venous access for percutaneous aspiration to enable quick initiation of cardiopulmonary bypass in case of an intraprocedural emergency.
